# The effects of AST-120 on chronic kidney disease progression in the United States of America: a *post hoc* subgroup analysis of randomized controlled trials

**DOI:** 10.1186/s12882-016-0357-9

**Published:** 2016-09-30

**Authors:** Gerald Schulman, Tomas Berl, Gerald J. Beck, Giuseppe Remuzzi, Eberhard Ritz, Miho Shimizu, Yuko Shobu, Mami Kikuchi

**Affiliations:** 1 Vanderbilt University School of Medicine, Nashville, TN USA; 2 University of Colorado Health Sciences Center, Denver, CO USA; 3 Cleveland Clinic Foundation, Cleveland, OH USA; 4 Unit of Nephrology and Dialysis, Azienda Ospedaliera Papa Giovanni XXIII, Bergamo, Italy; 5 IRCCS Istituto di Ricerche Farmacologiche Mario Negri, Bergamo, Italy; 6 Department of Biomedical and Clinical Sciences, University of Milan, Milan, Italy; 7 University of Heidelberg, Heidelberg, Germany; 8 Mitsubishi Tanabe Pharma Corporation, Tokyo, Japan; 9 Kureha Corporation, 3-26-2, Hyakunin-cho, Shinjuku-ku, Tokyo, 169-8503 Japan

**Keywords:** AST-120, Chronic kidney disease, Clinical trial, Spherical carbon adsorbent, Uremic toxin

## Abstract

**Background:**

The orally administered spherical carbon adsorbent AST-120 is used on-label in Asian countries to slow renal disease progression in patients with progressive chronic kidney disease (CKD). Recently, two multinational, randomized, double-blind, placebo-controlled, phase 3 trials (Evaluating Prevention of Progression in Chronic Kidney Disease [EPPIC] trials) examined AST-120’s efficacy in slowing CKD progression. This study assessed the efficacy of AST-120 in the subgroup of patients from the United States of America (USA) in the EPPIC trials.

**Methods:**

In the EPPIC trials, 2035 patients with moderate to severe CKD were studied, of which 583 were from the USA. The patients were randomly assigned to two groups of equal size that were treated with AST-120 or placebo (9 g/day). The primary end point was a composite of dialysis initiation, kidney transplantation, or serum creatinine doubling.

**Results:**

The Kaplan-Meier curve for the time to achieve the primary end point in the placebo-treated patients from the USA was similar to that projected before the study. The per protocol subgroup analysis of the population from the USA which included patients with compliance rates of ≥67 % revealed a significant difference between the treatment groups in the time to achieve the primary end point (Hazard Ratio, 0.74; 95 % Confidence Interval, 0.56–0.97).

**Conclusions:**

This *post hoc* subgroup analysis of EPPIC study data suggests that treatment with AST-120 might delay the time to primary end point in CKD patients from the USA. A further randomized controlled trial in progressive CKD patients in the USA is necessary to confirm the beneficial effect of adding AST-120 to standard therapy regimens.

**Trial registration:**

ClinicalTrials.gov NCT00500682; NCT00501046.

## Background

Treatment of end-stage renal disease (ESRD) is costly, and the prevalence of ESRD is increasing worldwide [1, 2]. The number of ESRD cases per year with diabetes as the primary cause has risen since 1980, with 50,000 ESRD cases in 2009 in the United States of America (USA) [2]. The prevalence of diabetes in ESRD cases in 2012 was 44 % [2]. Diabetic nephropathy occurs in 20–40 % of patients with diabetes and is the single leading cause of ESRD [3]. Clearly, preventing or slowing the progression of CKD and diabetes may reduce the cost of ESRD treatment considerably [4–7]. Current guidelines focus on managing factors that can hasten the progression of CKD, such as hypertension and proteinuria [8–10]. Although angiotensin-converting enzyme inhibitors (ACEIs) and angiotensin II receptor blockers (ARBs) slow the progression of CKD, many patients progress to ESRD despite taking these drugs. Because various pathologies contribute to the progression of CKD, additional interventions to decelerate the progression of CKD are needed.

AST-120 (Kureha Corporation, Tokyo, Japan) is an orally administered spherical carbon adsorbent approved in Japan for delaying the initiation of dialysis and ameliorating uremia symptoms in patients with progressive CKD [11, 12]. AST-120 has also been approved in Korea, the Philippines, and Taiwan. AST-120 reduces the concentrations of indoxyl sulfate (IS), a uremic toxin that enhances CKD progression, in the systemic circulation. AST-120 lowers IS levels by preventing the absorption of indole, a tryptophan breakdown product and a precursor of IS, from the gastrointestinal tract, which is the presumed mechanism underlying AST-120’s effect of slowing the progression of CKD [13].

Recently, two large, multinational, randomized, double-blind, placebo-controlled, phase 3 trials (Evaluating Prevention of Progression in Chronic Kidney Disease [EPPIC-1 and EPPIC-2]) were conducted to assess the effect of AST-120 in adults with CKD treated with standard therapies. The benefit of adding AST-120 to the standard therapy in patients with moderate to severe CKD was not supported by the results of the primary analysis of the intent-to-treat (ITT) population for each EPPIC trial or by the result of the pooled analysis of the two EPPIC trials [14]. We observed that disease progression in the EPPIC trial placebo population was more gradual than that estimated during study planning as reported previously [14]. In contrast, pre-specified subgroup analysis suggested that the population from the USA showed the expected primary event rate. Therefore, in this paper, we compared the end point rate and the disease progression among countries, and we conducted a *post hoc* subgroup analysis of the patients from the USA who were included in the EPPIC trials (EPPIC-USA), hypothesizing that they would be more likely to show a response to AST-120 administration.

## Methods

### Study design

Details of the EPPIC trials have been previously published including the patient flow diagram [14]. The protocols for the EPPIC trials were approved by local ethics committees, and the trials were conducted in accordance with Good Clinical Practice Guidelines of the International Conference on Harmonisation, the Declaration of Helsinki, and the European Union Clinical Trials Directive 2001/20/EC. The EPPIC trials were conducted between July 2007 and February 2012 at 239 international sites to compare the effects of AST-120 with those of placebo on renal outcomes in patients with moderate to severe CKD receiving standard therapy. Patients were randomly assigned to groups of equal size that were treated with 9 g/day AST-120 or placebo. AST-120 (provided as ten 300-mg capsules 3 times daily) or placebo was administered with meals and at least 1 h after concomitant medication. The trials were registered on ClinicalTrials.gov (NCT00500682 [EPPIC-1] and NCT00501046 [EPPIC-2]).

### Patients

Eligible patients were adults with moderate to severe CKD (defined as serum creatinine (sCr) at screening of 2.0–5.0 and 1.5–5.0 mg/dL for men and women, respectively) who were not expected to require dialysis or renal transplantation within 6 months of trial entry and who were expected to survive for ≥1 year. All patients were required to have proteinuria or progressive deterioration in renal function indicated by either a urinary total protein to urinary creatinine (UP/UCr) ratio >0.5 at screening or an sCr level that had increased by >10 % at a second evaluation conducted 3 months after screening.

### Outcomes

The primary end point was a triple composite of the time from the date of group assignment to the date of kidney disease progression, as indicated by dialysis initiation, kidney transplantation, or doubling of the sCr level, whichever occurred first.

### Statistical analysis

The pre-specified statistical methods used for the EPPIC trials’ analyses were applied in the same manner for these *post hoc* subgroup analyses. A pooled population of both EPPIC trials was used for all analyses. The primary end point was analyzed using a stratified Cox proportional hazards regression model with a 95 % confidence interval (CI), and Kaplan-Meier (K-M) methodology was used to calculate the median time from group assignment to renal disease onset. The unstratified Cox regression method was used to analyze the individual components of the composite end point. The covariate adjustments were CKD etiology (diabetic or non-diabetic nephropathy) and baseline sCr level (greater than or less than 3 mg/dL) in all analysis, and region (North America, Latin America, or Europe) was added for the all country primary analysis The unstratified Cox regression method was used to analyze the primary end point within the following baseline subgroups: diabetic nephropathy, sCr level, CKD stage determined by the estimated glomerular filtration rate (eGFR) level, C-reactive protein (CRP) level, anemia status, age, race, gender, use of ACEI or ARB at baseline, and UP/UCr ratio. Declines in eGFR were calculated for the first 96-week treatment period to elucidate the degree of renal disease progression.

The ITT population included all randomly assigned patients who received at least one dose of the study drug and had at least one post-baseline sCr measurement. The per protocol (PP) population included all patients in the ITT population without major protocol violations which were: receiving treatment other than that originally assigned, having a treatment compliance rate of <67 %, and/or a treatment period of <8 weeks. Inclusion in the PP population was determined during a blinded data review conducted prior to locking of the database and study unblinding. Patients who did not reach the primary end point were censored at the date of last contact. Sensitivity analyses of the primary efficacy end point were performed to evaluate the robustness of the results to censoring patterns.

## Results

The number of patients, primary end point rates, median time to the primary end point, eGFR decline, and eGFR at dialysis initiation by country are summarized in Table 1. While the median time to the primary end point pre-study was estimated to be 124 weeks, the actual time for the entire population was 180.1 weeks. The median time to the primary end point could be calculated only for patients from the USA (135.6), Argentina (135.7), Germany (119.3), and Spain (71.3). Of the countries for which the analysis was possible, the USA had the largest study population (AST-120, *N* = 290; placebo, *N* = 293).

**Table 1:** Primary efficacy end point achievement and disease progression by country (pooled ITT population)

Country		AST-120		Placebo		Placebo		
		*N*	*n* (%)	*N*	*n* (%)	Median Time ^a^	eGFR decline ^b^	eGFR at Dialysis Initiation ^c^
ALL		1000	350 (35.0)	999	360 (36.0)	180.1	−4.96 ± 9.50	10.47 ± 5.28
NA	CAN	61	22 (36.1)	57	19 (33.3)	ND	−6.25 ± 9.56	10.65 ± 3.28
	USA	290	120 (41.4)	293	129 (44.0)	135.6	−4.66 ± 11.42	13.95 ± 5.84
LA	ARG	73	20 (27.4)	73	27 (37.0)	135.7	−5.17 ± 7.69	9.15 ± 2.47
	BRA	84	26 (31.0)	83	25 (30.1)	ND	−3.13 ± 6.65	8.82 ± 3.39
	MEX	58	21 (36.2)	57	18 (31.6)	ND	−5.13 ± 8.35	7.45 ± 3.57
EU	CZE	21	6 (28.6)	20	5 (25.0)	ND	−3.52 ± 3.02	11.91 ± 1.86
	DEU	7	2 (28.6)	8	4 (50.0)	119.3	−3.49 ± 14.93	14.77 ± 3.29
	ESP	8	3 (37.5)	9	5 (55.6)	71.3	−4.20 ± 4.12	9.08 ± 1.24
	FRA	17	5 (29.4)	15	4 (26.7)	ND	−3.93 ± 7.59	11.24 ± 2.68
	ITA	8	4 (50.0)	7	2 (28.6)	ND	−2.60 ± 2.60	8.18 ± 1.80
	POL	36	18 (50.0)	38	15 (39.5)	ND	−3.50 ± 6.97	10.00 ± 3.23
	RUS	173	57 (32.9)	174	51 (29.3)	ND	−4.78 ± 7.73	7.32 ± 2.90
	UKR	164	46 (28.0)	165	56 (33.9)	ND	−7.17 ± 10.55	5.72 ± 2.67

^a^Weeks, ^b^mL/min/1.73 m^2^/year, ^c^mL/min/1.73 m^2^

*ND* Not detected

*NA* North America, *LA* Latin America, *EU* Europe, *CAN* Canada, *USA* United States of America, *ARG* Argentina, *BRA* Brazil, *MEX* Mexico, *CZE* Czech Republic, *DEU* Germany, *ESP* Spain, *FRA* France, *ITA* Italy, *POL* Poland, *RUS* Russia, *UKR* Ukraine

**Fig. 1:**
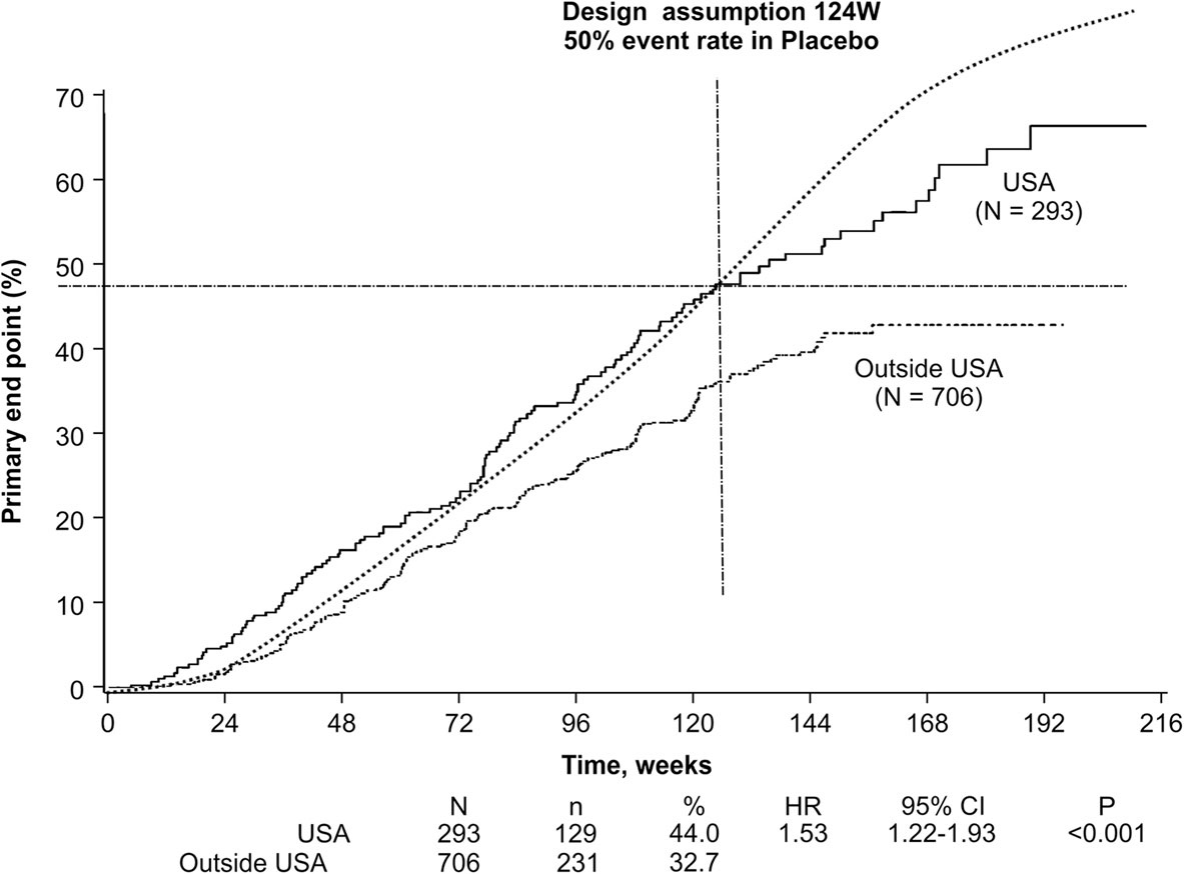
Kaplan-Meier plots of primary end point achievement in placebo-treated USA and outside USA populations. *CI* confidence interval, *HR* hazard ratio

The K-M curve for time to the primary end point for placebo-treated EPPIC-USA patients was similar to the curve estimated during study planning, while the curve for the placebo-treated non-USA patients differed significantly, as shown in Fig. 1. Therefore, we selected the EPPIC-USA population to assess the effect of AST-120 in patients with CKD.

Demographics and baseline clinical characteristics of EPPIC-USA patients are shown in Table 2. Sixty percent of the EPPIC-USA patients were diabetic and 80 % of the EPPIC-USA patients were taking an ACEI or an ARB at baseline data collection. There were no significant differences in demographics and baseline clinical characteristics between the AST-120 and placebo groups. We applied the pre-specified statistical methods used in the EPPIC trials [14] to the EPPIC-USA population.

**Table 2:** Demographic and baseline clinical characteristics of the EPPIC-USA population (pooled ITT population)

	AST-120	Placebo	*P*-value
	*N* = 290	*N* = 293	
Age, years, mean ± SD	60.1 ± 14.3	61.7 ± 12.3	0.15
Sex, %			
Male	64.5	70.6	0.11
Race, %^a^			
White	66.6	60.8	0.51
Black or African American	19.7	25.3	
Asian	6.2	5.1	
Native Hawaiian or other Pacific Islander	0.7	0.7	
American Indian or Alaska Native	0.0	0.3	
Other	6.9	7.8	
CKD etiology, %			
Diabetic nephropathy	58.6	61.8	0.44
Type I Diabetes	6.2	3.4	
Type II Diabetes	52.4	58.4	
Non-diabetic nephropathy	41.4	38.2	
Glomerulonephritis	7.6	12.3	
Nephrosclerosis	16.2	15.7	
Other	17.6	10.2	
Use of ACEI or ARB, %			
Yes	79.7	80.5	0.79
Baseline sCr, mg/dL, mean ± SD^b^	3.01 ± 0.84	3.08 ± 0.80	0.28
Baseline eGFR, mL/min/1.73 m^2^, mean ± SD	23.59 ± 7.79	23.04 ± 6.92	0.36
Baseline UP/UCr ratio			
*N*	288	292	0.06
Mean ± SD	1.94 ± 1.29	2.15 ± 1.40	
CKD stage, %			
Stage 3a	0.3	0.7	0.70
Stage 3b	20.7	14.3	
Stage 4	64.5	74.4	
Stage 5	14.5	10.6	
Baseline anemia status, %^c^			
Yes	76.2	79.9	0.25
BMI, kg/m^2d^			
*N*	289	293	0.20
Mean ± SD	31.8 ± 7.2	32.7 ± 8.7	

^a^Race was self-reported

^b^To convert sCr from mg/dL to mol/L, multiply by 88.4

^c^Anemia was defined as a hemoglobin level <13.5 g/dL (men) or <12.0 g/dL (women)

^d^Body mass index is the weight in kilograms divided by the square of the height in meters

**Fig. 2:**
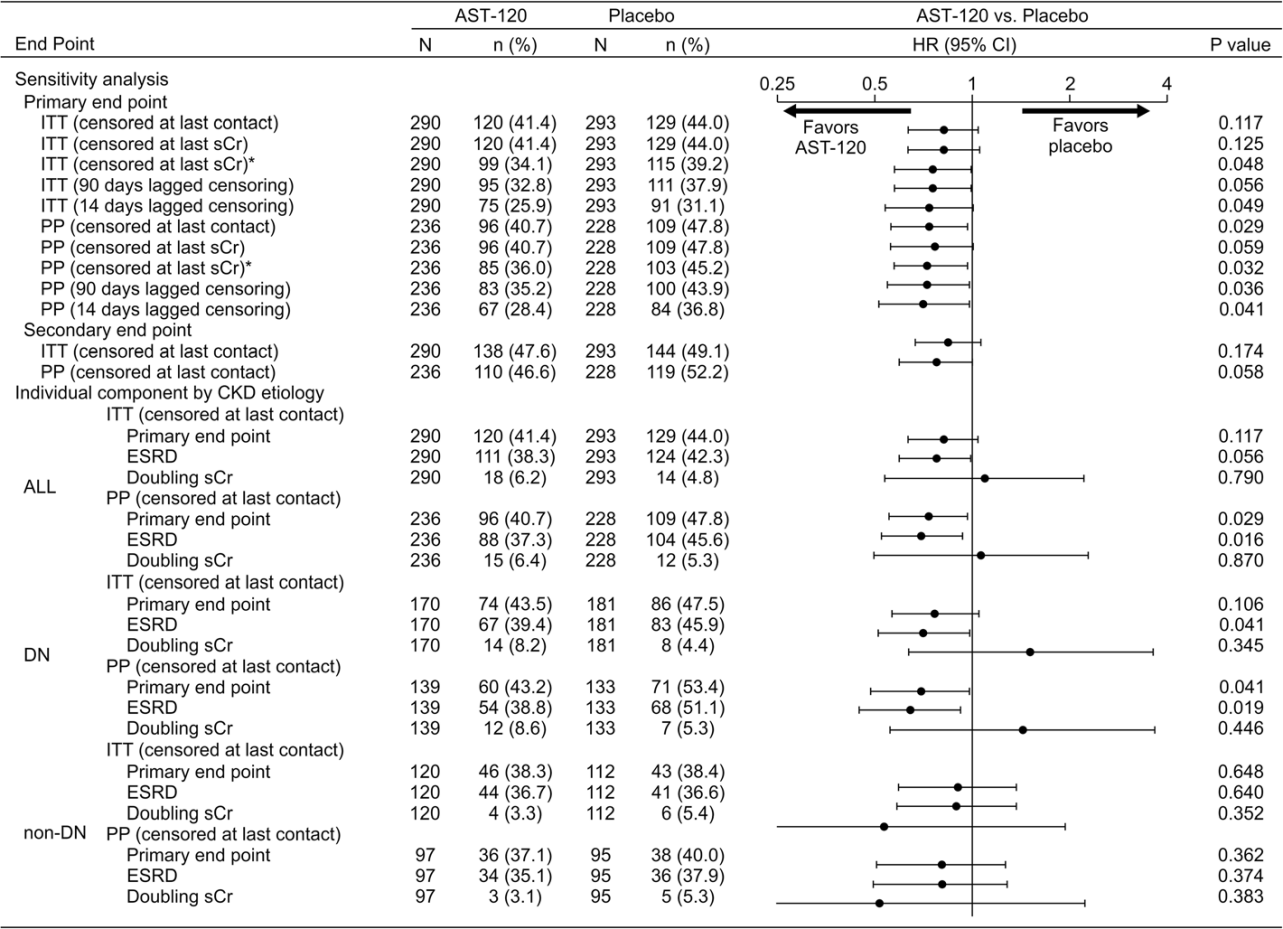
Achievement of primary and secondary end points in the EPPIC-USA subpopulation. ^a^ Sensitivity analyses of the primary efficacy end point were performed to evaluate the robustness of the results to censoring patterns. ^b^ First occurrence of dialysis, kidney transplantation, or doubling of sCr in the 84 days after the last sCr assessment or last dose. Patients who did not have an event in this period were censored at the last sCr assessment. *CKD* chronic kidney disease, *DN* diabetic nephropathy, *ESRD* end stage renal disease, *HR* hazard ratio, *ITT* intent-to-treat, *PP* per protocol, *sCr* serum creatinine

**Fig. 3:**
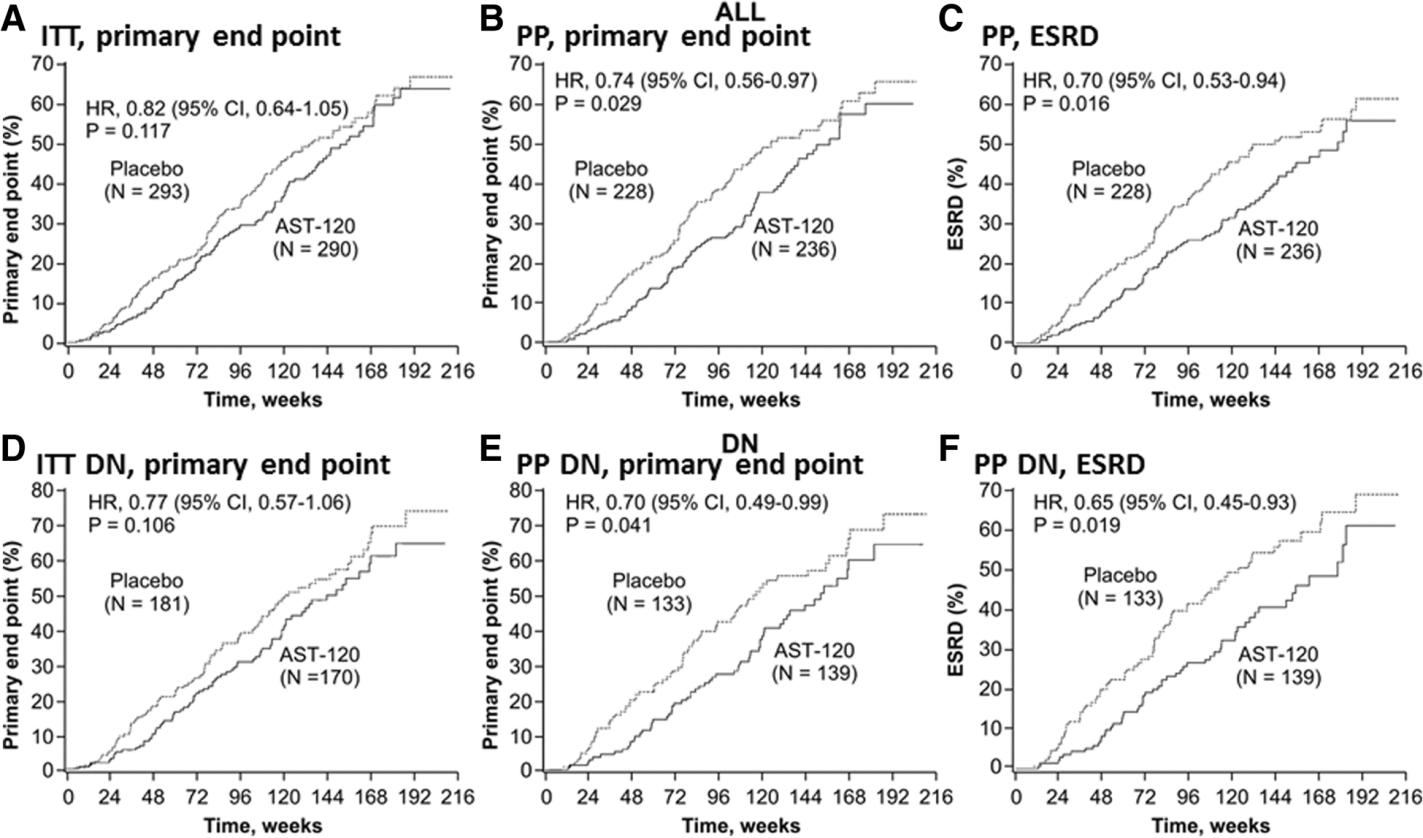
Kaplan-Meier plots of primary end point achievement in the EPPIC-USA population. **a** ITT primary end point censored at last contact, **b** PP primary end point censored at last contact, **c** PP ESRD censored at last contact, **d** ITT DN primary end point censored at last contact, **e** PP DN primary end point censored at last contact, **f** PP DN ESRD censored at last contact. *DN* diabetic nephropathy, *ESRD* end stage renal disease, *ITT* intent-to-treat, *PP* per protocol

The results of the primary and secondary analyses, including the sensitivity analyses, are shown in Fig. 2. While there was no significant difference in primary end point achievement between the treatment arms in the EPPIC-USA ITT population (hazard ratio (HR), 0.82; 95 % CI, 0.64–1.05; *P* = 0.117), treatment with AST-120 reduced the risk of achieving the primary end point in the PP population censored at last contact (HR, 0.74; 95 % CI, 0.56–0.97; *P* = 0.029). K-M curves for the ITT and PP populations are shown in Fig. 3a and b, respectively.

In the EPPIC-USA population, the majority of the primary end points were ESRD (defined as dialysis and transplantation), while doubling of sCr occurred infrequently. In the PP population, treatment with AST-120 reduced the risk of achieving ESRD (HR, 0.70; 95 % CI, 0.53–0.94; *P* = 0.016) (Fig. 2). The K-M curves for these results are shown in Fig. 3c.

In the subgroup of patients with diabetic nephropathy indicated as the CKD etiology, there was a significant difference in the time to primary end point (HR, 0.70; 95 % CI, 0.49–0.99; *P* = 0.041) and the time to ESRD (HR, 0.65; 95 % CI, 0.45–0.93; *P* = 0.019) between the AST-120 and placebo groups in the PP population, but there were no significant differences in the time to primary end point in the diabetic nephrology ITT population (HR, 0.77; 95 % CI, 0.57–1.06; *P* = 0.106) (Fig. 2). The K-M curves for these results are shown in Fig. 3e, f, and d, respectively. These results were similar to that of the whole population (Fig. 2, ALL).

**Fig. 4:**
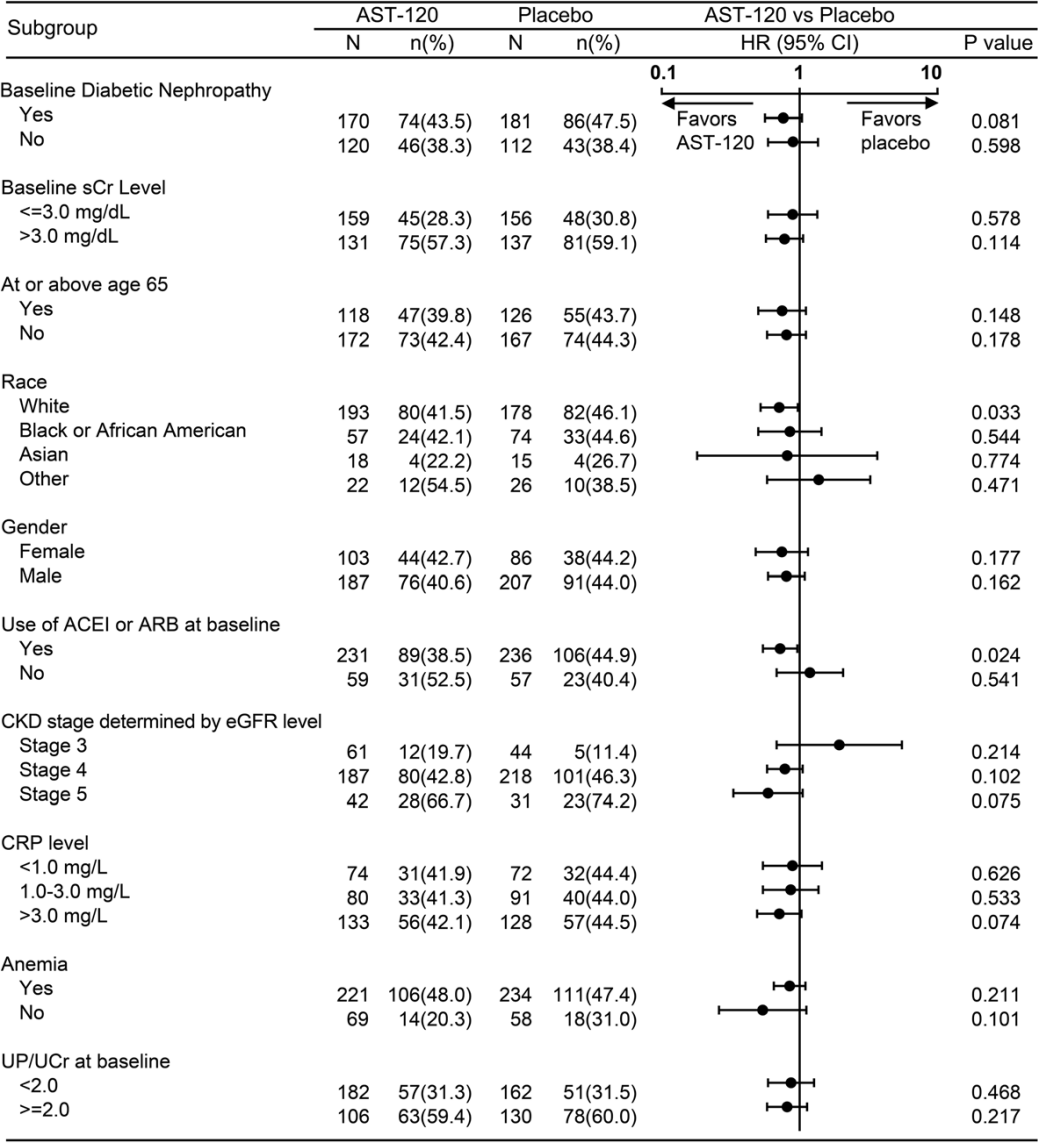
Achievement of primary end points in the EPPIC-USA subgroups. *ACEI* angiotensin converting enzyme inhibitor, *ARB* angiotensin receptor blocker, *CKD* chronic kidney disease, *CRP* C-reactive protein, *eGFR* estimated glomerular filtration rate, *sCr* serum creatinine, *UP/UCr* Urinary total protein to urinary creatinine ratio

The results of the subgroup analysis are shown in Fig. 4. Treatment with AST-120 reduced the risk of achieving the primary end point in the white (in race) and ACEI/ARB treatment-receiving patient subgroup. A tendency was observed that AST-120 treatment delays the time to primary end point in patients with DN, with CKD stage 5 and CRP levels (>3.0 mg/L). No effect was observed in other baseline parameters and demographic characteristics including age and gender.

## Discussion

This *post hoc* analysis of EPPIC studies in CKD patients from 13 countries demonstrated that the occurrence of primary end point, that is the composite of dialysis initiation, kidney transplantation, and serum creatinine doubling, was different among countries. Particularly, the time to primary end point in the EPPIC-USA placebo population was shorter than that of the placebo population outside the USA (*P* < 0.001). The incidence rates of ESRD continue to become clear based on the enhancement of renal databases in each countries and regions. However, the international difference of CKD progression and time to ESRD of CKD patients have not well discussed and remain unclear.

Recently, the National Kidney Foundation (NKF) and Food and Drug Administration (FDA) published a series of reports showing that eGFR decline associated with ESRD occurrence [15–18]. However, the value of eGFR decline in the EPPIC-USA placebo population did not differ from that in EPPIC overall population. The time to end point achievement by each country was not related to the observed eGFR decline in the EPPIC population. Thus, the difference in ESRD occurrence among the countries could not be attributed to differences in eGFR decline. Other possible causes may be assumed as following.

First, regional differences in the mean eGFR at dialysis could have contributed to the time to primary end point. Of the countries participating in the EPPIC trials, the USA had the highest mean eGFR at dialysis (13.53 ± 5.68 mL/min/1.73 m^2^), which was close to the K/DOQI criterion of 15 mL/min/1.73 m^2^ [19]. This observation is consistent with data from the US Renal Data System (USRDS), which shows that >15 % of patients started dialysis with an eGFR of ≥15 mL/min/1.73 m^2^ after 2009 [2]. The median eGFR at the start of dialysis was 7.7 mL/min/1.73 m^2^ in the 2003 European Renal Association-European Dialysis and Transplant Association Registry [20], notably lower than that in USRDS. Regional differences may account for the observation that the majority of the primary end points were ESRD in the EPPIC-USA population.

Second, the dietary habit and nutritional status are variety among countries and it has been proposed that they could affect the CKD progression. The role of nutrition in the progression of CKD is unclear with discrepancies between various studies performed, but some reviews suggested that dietary protein intake [21] and obesity [22] are risk factors for CKD. In EPPIC studies, nutritional information was not collected. However the demographic parameter of patients showed that the mean of body mass index (BMI) and diabetes ratio of USA patients was higher than that of patients outside the USA at baseline (p <0.001). These could relate to the time to renal events.

The difference in event rates observed between countries may have also been due to the influence of differing healthcare standards and guidelines. Prior to study start, we estimated the primary end point rate for a placebo population based on RENAAL study data; in that study, 45 % of study patients were from the US [23]; we found the median time to primary end point of EPPIC-USA placebo population was similar to the estimated median time of placebo population in the RENNAL study.

A trend for a beneficial effect of AST-120 was observed in the EPPIC-USA PP population comprised of patients who took the study medication as prescribed, had study drug compliance rates ≥67 %, and were treated for at least 8 weeks. This effect was corroborated by sensitivity analyses that assessed the time lag between treatment cessation and event occurrence. Therefore, good patient compliance highly determines the positive effect of AST-120 in preventing systemic accumulation of uremic toxins, an important factor considering the 30 capsule per day regimen. The difficulty of AST-120 medication may be an issue needing to be solved as relatively large number of capsule should be taken. AST-120 treatment was beneficial in patients with diabetic nephropathy; however, for patients with an etiology other than diabetic nephropathy, there was no difference between the AST-120-treated and placebo groups. We speculate that the difference in etiology is responsible for the difference in event rates between the placebo groups with diabetic nephropathy and non-diabetic nephropathy (47.5 vs. 38.4 %, respectively). Results from an additional subgroup analysis in ACEI- or ARB-treated patients suggested that the addition of AST-120 to standard therapy produced therapeutic benefits by a distinct mechanism of action that involved lowering levels of uremic toxins such as IS [24].

In addition, examining the use of AST-120 in populations that have a particular rate of disease progression, such as diabetic nephropathy patients or ESRD patients with residual renal function, may be useful. Atoh et al. reported a strong correlation between IS serum levels and eGFRs [25]. The EUTox group reported that IS serum levels were increased significantly and progressively as patients progressed through the stages of CKD, including predialysis and hemodialysis patients [26].

## Conclusions

This *post hoc* subgroup analysis of EPPIC study data suggests that treatment with AST-120 might delay the time to primary end point in CKD patients from the USA. The major limitations of this analysis are its *post hoc* nature and its use of one subgroup population. The conclusions of this study are hypothesis-generating only, and the *p*-values should not be interpreted by the usual standards regarding a Type-1 error threshold of 0.05. According to our findings, a randomized controlled trial in progressive CKD patients in the USA is necessary to confirm the beneficial effect of adding AST-120 to standard therapy regimens.

## Abbreviations

ACEIs: Angiotensin-converting enzyme inhibitors
ARBs: Angiotensin II receptor blockers
CI: Confidence interval
CKD: Chronic kidney disease
CRP: C-reactive protein
DN: Diabetic nephropathy
eGFR: Estimated glomerular filtration rate
EPPIC: Evaluating Prevention of Progression in Chronic Kidney Disease trials
EPPIC-USA: Patients from the USA who were included in the EPPIC trials
ESRD: End-stage renal disease
HR: Hazard ratio
IS: Indoxyl sulfate
ITT: Intent-to-treat
K-M: Kaplan-Meier
PP: Per protocol
sCr: Serum creatinine
UP/UCr: Urinary total protein to urinary creatinine ratio
USA: United States of America
USRDS: US Renal Data System
